# Perioperative and Mid-term Outcomes of the Ankura Thoracic Stent Graft in Aortic Arch Pathologies With Open and Endovascular Perfusion of Supra-Aortic Branches: A Retrospective Review

**DOI:** 10.7759/cureus.88594

**Published:** 2025-07-23

**Authors:** Konstantinos Tigkiropoulos, Georgios Chatziantoniou, Katerina Sidiropoulou, Alexandros Apostolou, Kyriakos Stavridis, Dimitrios A Chatzelas, Dimitrios Karamanos, Georgios Pitoulias, Nikolaos Saratzis

**Affiliations:** 1 Division of Vascular Surgery, 1st Surgical Department, School of Medicine, Aristotle University of Thessaloniki, Papageorgiou General Hospital, Thessaloniki, GRC; 2 Division of Vascular Surgery, 2nd Department of Surgery, School of Health Sciences, Aristotle University of Thessaloniki, General Hospital of Thessaloniki "G. Gennimatas", Thessaloniki, GRC

**Keywords:** ankura thoracic stent graft, aortic arch, chimney, extra-anatomic bypass, tevar

## Abstract

Background and aims: This study reports on the perioperative and mid-term outcomes of a novel thoracic stent graft in aortic arch pathologies.

Methods: From January 2017 to May 2023, all patients treated with Ankura (Lifetech Scientific, Shenzhen, China) thoracic endograft combined with extra-anatomic and/or endovascular perfusion of supra-aortic branches at two university vascular centers were retrospectively reviewed. Indications for treatment included aneurysm, dissection, penetrating ulcer, intramural hematoma, traumatic rupture of the aortic arch and endoleak type Ia (Ia EL) of previous endovascular repair of the descending thoracic aorta. Perioperative and late causes of morbidity and mortality were captured. Patients' follow-up was obtained by computed tomography angiography at one month, six months, one year and then annually. The primary endpoint of the study was technical success, and secondary endpoints included aortic-related complications and all-cause mortality during follow-up.

Results: Aortic arch repair with Ankura thoracic endograft was performed in 30 patients. Mean age was 69.3 years (53-85 years), and male was the prevalent gender (25 patients, 83%). According to Ishimaru classification, Ankura stent graft was deployed at zone 2 in 21 patients (70%), at zone 1 in eight patients (26.6%) and at zone 0 in one patient (3.3%). Technical success was 94%. Type Ia EL was detected at two patients (6.6%). Perioperative morbidity and mortality were 6.6% and 3.3%, respectively. Mean follow-up was 22.03 months (range: 1-24 months). The aortic reintervention rate was 0%, and all-cause mortality was 16.6%.

Conclusion: Ankura thoracic endograft showed satisfactory results with high technical success and low aortic-related complications in our study. Larger studies are necessary to establish the safety and efficacy of Ankura endograft in aortic arch pathologies.

## Introduction

Aortic arch pathology is a challenging area for cardiac and vascular surgeons due to the complexity of the anatomy and the presence of supra-aortic branches. Open surgical repair with cardiopulmonary bypass and deep hypothermic arrest was considered for decades the primary treatment option [1]. However, it was associated with significant perioperative risks [1]. Thoracic endovascular aortic repair (TEVAR) was introduced in 1994 by Dake, and nowadays, it is considered the primary treatment modality to manage all types of descending thoracic aortic pathology, including aneurysm, dissection, intramural hematoma, penetrating ulcer and traumatic rupture [2,3]. With evolution of endovascular techniques, advanced imaging and planning and careful patient selection, the indications for TEVAR have expanded, to treat lesions in the aortic arch using branched, fenestrated, chimney and hybrid techniques with acceptable perioperative results minimizing neurologic complications [4-8]. One of the novel thoracic stent grafts is Ankura (Lifetech Scientific, Shenzhen, China) which received Conformite Europeenne (CE) Mark in 2011. The aim of this study is to present the efficacy as well as to report the perioperative and mid-term outcomes of patients with aortic arch pathologies treated with Ankura thoracic stent graft.

## Materials and methods

This retrospective study from two university vascular centers of Northern Greece analyzed the outcomes of 30 patients who underwent endovascular repair of the aortic arch with Ankura thoracic stent graft (Lifetech Scientific, Shenzhen, China) between January 2017 and May 2023. Informed consent was obtained from all patients. Ethical approval from the local Committees was waived off due to the retrospective nature of the study. Informed consent was obtained from all patients involved in the study before the procedure. Inclusion criteria were males and females older than 18 years old with aortic diseases originating in the aortic arch and patients with endoleak type Ia (Ia EL) of previous TEVAR that necessitate proximal extension to the aortic arch (Table 1).

**Table 1:** Indications for endovascular repair of aortic arch lesions with thoracic Ankura stent graft. TEVAR: thoracic endovascular aortic repair.

Dissection
Fusiform/saccular aneurysm
Penetrating ulcer
Traumatic rupture
Intramural hematoma
Type Ia endoleak of previous TEVAR

The medical history of the patients and perioperative outcomes were recorded. Patients' follow-up included clinical examination at outpatient clinic and computed tomography angiography at one month, six months, one year and then annually postoperatively. Baseline characteristics of patients are presented in Table 2.

**Table 2:** Demographics and baseline characteristics of the study group.

Gender/concomitant diseases	Number of patients/percentage
Men	25/30 (83%)
Carotid disease	1/30 (3.3%)
Coronary artery disease	10/30 (33.3%)
Hypertension	26/30 (86.6%)
Diabetes mellitus	7/30 (23.3%)
Dyslipidemia	17/30 (56.6%)
Cancer	4/30 (13.3%)
Chronic obstructive pulmonary disease	1/30 (3.3%)
Atrial fibrillation	2/30 (6.6%)
Chronic renal failure	1/30 (3.3%)

Device characteristics

**Figure 1:**
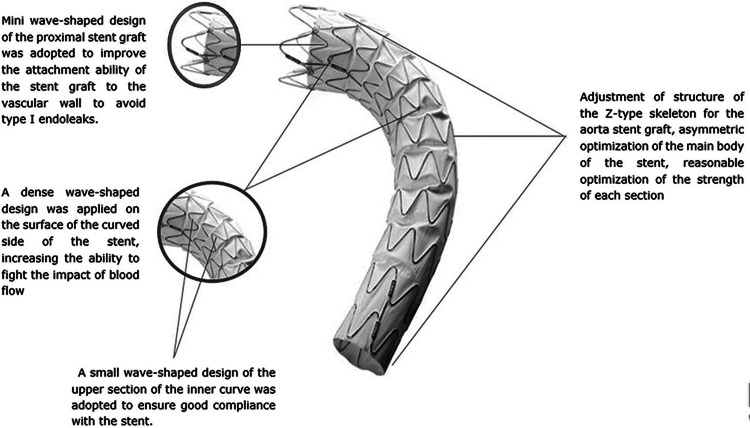
Device characteristics of Ankura thoracic stent graft. From Shu et al. [10].

The Ankura thoracic stent graft is composed of a dual layer expanded polytetrafluoroethylene membrane with a proximal bare stent. There is no suture in the main body to avoid pinhole leakage providing better durability. The proximal part of the graft has mini wave-shaped design to provide better apposition of the graft to the aortic wall, whereas dense wave design is on the curve side of the graft to fight blood flow and to avoid distal migration [9,10]. The connecting bar of the graft must be on the outer curve of the aortic arch having the same side as the hemostatic valve before insertion to the access vessel. In case of tortuous anatomy of aorta and iliac vessels, hemostatic valve may not be on the same side with the connecting bar. The 8-shaped marker on the proximal part of the endograft ensures the position of the connecting bar to the outer curve of aortic arch (Figure 1).

Operative procedure

All procedures were performed in a hybrid operating room under fluoroscopic guidance. The type of the procedure (hybrid or endovascular) was based on the anatomic characteristics of supra-aortic branches and on surgeon’s discretion. After induction to general anesthesia, arterial access was gained via surgical cutdown of one common femoral artery according to our clinic's protocol and the patient was systematically heparinized to achieve an activated clotting time (ACT) between 230 and 300 sec. A Lunderquist extra-stiff guidewire (William Cook Europe, Bjaeverskov, Denmark) was advanced into ascending aorta. Perfusion of the supra-aortic branches was performed either endovascularly (chimney technique and in situ fenestration with Fu-through needle catheter (Lifetech Scientific, Shenzhen, China)) after surgical cutdown of the access artery (left brachial artery and/or left common carotid artery) or by open surgical technique (Carotid-subclavian bypass) with Propaten synthetic graft (W.L. Gore & Associates, Flagstaff, AZ, United States). Oversizing of the Ankura graft was 30% in chimney technique. In case of endovascular repair of dissection and traumatic rupture without chimney technique, oversizing was 0%-10% and 20% for aneurysm, penetrating ulcer and intramural hematoma. The overlapping length of aortic and chimney grafts was more than 2cm. Chimney stent grafts were balloon-covered stents, including Lifestream (B.D Corp, Inc, Murray Hill, NJ, United States), VBX (W.L. Gore & Associates, Flagstaff, AZ, United States) and iCover (IVascular, Barcelona, Spain) which were oversized by 1mm compared to the diameter of the supra-aortic branch. The Ankura stent graft was deployed in the targeted position under pharmacologically induced hypotension (systolic pressure 70-80mmHg), and the chimney stent grafts were inserted from the brachial/left carotid artery and deployed parallel to the aortic endograft. Digital subtraction angiography (DSA) was performed to verify exclusion of aortic pathology, type Ia endoleak and patency of supra-aortic branches. In the presence of EL 1a, kissing balloon was performed with Reliant molding balloon (Medtronic, Inc, Minneapolis, MN, United States) and Ultraverse balloon (B.D Corp, Inc, Murray Hill, NJ, United States) to the stent grafts. Postoperatively patients were prescribed Aspirin 100mg and low-molecular-weight heparin in prophylactic dose during hospitalization, followed by lifelong single antiplatelet regimen after discharge.

Endpoints

The primary endpoint of the study was technical success which is defined as successful deployment of the Ankura stent graft at the targeted lesion with complete exclusion of aortic pathology, absence of endoleak type I and II and patency of supra-aortic branches at final angiography. Secondary endpoints were aortic-related complications (endoleak type I, II, patency of branches) and all-cause mortality during follow-up.

Statistical analysis

Statistical analysis was performed using SPSS software program (SPSS version 29; IBM corporation, Armonk, NY). Data are expressed as means for continuous variables and counts (%) for nominal variables. The Kaplan-Meier survival analysis was used to establish all-cause mortality of the study group.

## Results

**Figure 2:**
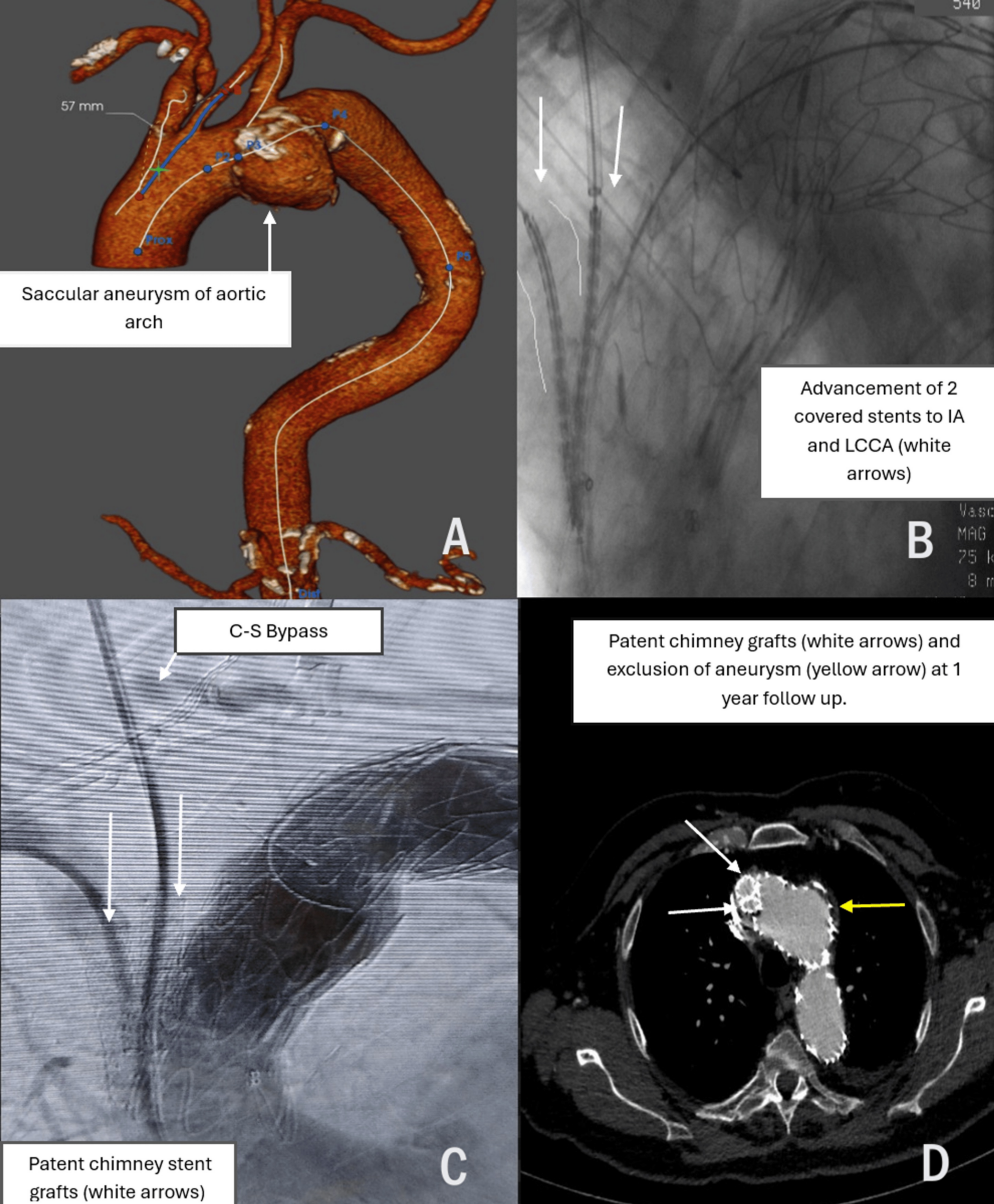
Saccular aneurysm of the aortic arch treated with double chimney stents and carotid-subclavian bypass. A. A male patient (77 years old) with a saccular aneurysm of the inner curve of aortic arch. B. Double chimney to innominate and left common carotid artery. C. Final angiography depicted exclusion of aneurysm with patent chimney stent grafts and left carotid-subclavian bypass (C-S bypass). D. Computed tomography angiography follow-up at one year showed exclusion of aneurysm and patent chimney-covered stents. LCCA: left common carotid artery.

Ankura stent graft was totally deployed in 30 patients (men n=25, 83%) with a mean age of 69.3 years (53-85). Technical success was 94%. Two patients underwent TEVAR in an emergent setting (one patient with a traumatic rupture after a car accident, and one patient with persistent thoracic pain due to intramural hematoma). Seventeen patients had a single chimney at the left subclavian artery (LSA), and four patients had a double chimney to the left common carotid artery (LCCA) and LSA. Two patients had Kommerell diverticulum who underwent TEVAR with bilateral carotid-subclavian bypass and plugs at both subclavian arteries. One patient had a double chimney at innominate-LCCA and carotid-subclavian bypass due to a saccular aneurysm at the middle part of the aortic inner curve (Figures 2A-2D).

**Figure 3:**
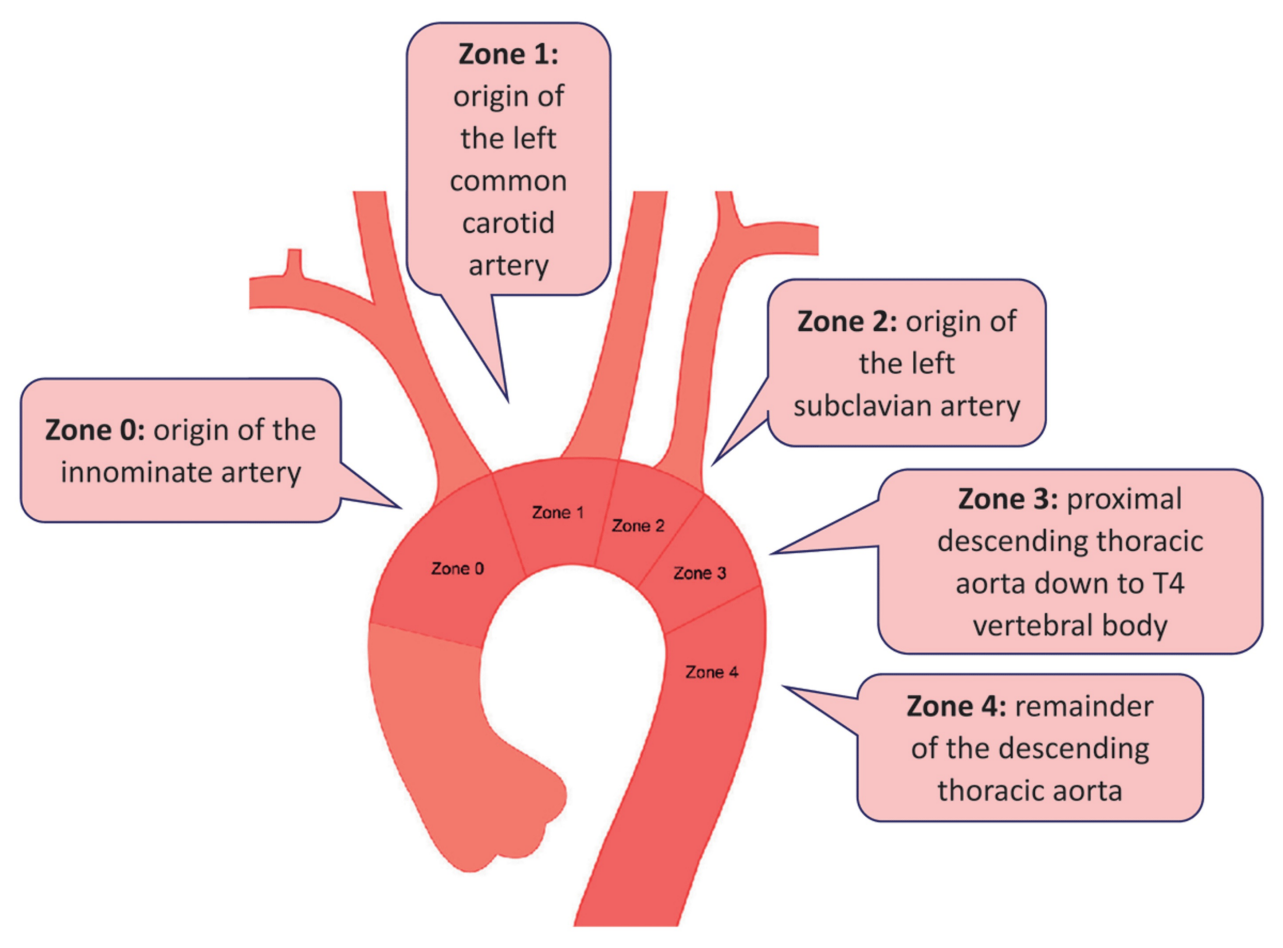
The Ishimaru classification scheme defines the five TEVAR landing zones for comparing endovascular procedures. Each zone is bordered by a tangential line aligned with the distal edge of each great vessel, including zone 0, the origin of the innominate artery; zone 1, the origin of the left common carotid artery; zone 2, the origin of the left subclavian artery; zone 3, the proximal descending thoracic aorta down to T4 vertebral body; and zone 4, the remainder of the descending thoracic aorta. TEVAR: thoracic endovascular aortic repair. From Atkins et al. [11].

LCCA chimney with in situ needle fenestration of LSA with Fu-through needle catheter (Lifetech Scientific, Shenzhen, China) was performed in one patient. Two patients had previous TEVAR due to descending thoracic aortic aneurysm. They developed type Ia endoleak due to evolution of the aneurysm at the proximal landing zone. They successfully managed with proximal stent graft extension and chimney to LSA. According to Ishimaru classification (Figure 3) [11], Ankura stent graft was deployed at zone 2 in 21 patients (70%), at zone 1 in eight patients (26.6%) and at zone 0 in one patient (3.3%).

Type Ia EL was detected at delayed phase of final angiography in two patients with distal aortic arch aneurysm and single chimney of LSA which were managed conservatively. Table 3 summarizes the type of procedures, aortic pathology and proximal landing zone of the study group.

**Table 3:** Types of procedures, aortic arch pathology and proximal landing zone of Ankura thoracic endograft in the study group. EL: endoleak, PLZ: proximal landing zone, LSA: left subclavian artery, LCCA: left common carotid artery, IA: innominate artery, C-S: carotid-subclavian, M: male, F: female, TEVAR: thoracic endovascular aortic repair, IMH: intramural hematoma.

Patient	Gender	Age	Aortic pathology	Type of procedure	PLZ
Patient 1	M	68	Aneurysm	Chimney LCCA + C-S bypass	1
Patient 2	M	59	Penetrating ulcer/IMH	Chimney LSA	2
Patient 3	F	72	Aneurysm	Chimney LSA	2
Patient 4	M	73	Aneurysm	C-S bypass + plug	2
Patient 5	M	64	Kommerell diverticulum	(2x) C-S bypass + plugs	2
Patient 6	M	85	Aneurysm	Chimney LSA	2
Patient 7	F	81	Aneurysm	Chimney LSA	2
Patient 8	M	77	Aneurysm	Chimney IA-LCCA + C-S bypass	0
Patient 9	M	66	Aneurysm	Chimney LCCA-LSA	1
Patient 10	M	69	Aneurysm	Chimney LCCA-LSA	1
Patient 11	M	70	Aneurysm	Chimney LCCA + in situ fenestration LSA	1
Patient 12	M	59	Dissection	Chimney LSA	2
Patient 13	F	55	Post-traumatic aneurysm	Chimney LSA	2
Patient 14	F	78	Aneurysm	C-S bypass + plug	2
Patient 15	M	60	Aneurysm	Chimney LCCA + C-S bypass + plug	1
Patient 16	M	55	Aneurysm	C-S bypass + plug	2
Patient 17	M	70	Aneurysm	Chimney LCCA-LSA	1
Patient 18	M	73	Dissecting aneurysm	Chimney LSA	2
Patient 19	M	68	Penetrating ulcer	Chimney LCCA-LSA	1
Patient 20	F	81	Aneurysm	Chimney LSA	2
Patient 21	M	78	EL1 TEVAR aneurysm	Chimney LSA	2
Patient 22	M	59	EL1 TEVAR aneurysm	Chimney LSA	2
Patient 23	M	53	Dissection	Chimney LSA	2
Patient 24	M	72	Penetrating ulcer	Chimney LSA	2
Patient 25	M	75	Aneurysm	Chimney LSA	2
Patient 26	M	68	Dissection	Chimney LSA	2
Patient 27	M	74	Aneurysm	Chimney LSA	2
Patient 28	M	59	Kommerell diverticulum	C-S bypass + Chimney LCCA + plug	1
Patient 29	M	63	Dissection	Chimney LSA	2
Patient 30	M	72	Traumatic rupture	Chimney LSA	2

No conversion or additional endovascular procedure was performed during perioperative period. Two patients had procedure-related complications. One patient experienced stroke after operation (right hemiparesis) probably due to thromboembolism in the presence of a severe atheromatous disease of aortic arch. The second patient had axillary plexus injury after carotid-subclavian bypass with minor motor disturbances of the left upper limb. One patient died on the first postoperative day due to myocardial infarction. The 30-day morbidity and mortality in the group was 6.6% and 3.3%, respectively.

Follow-up

**Figure 4:**
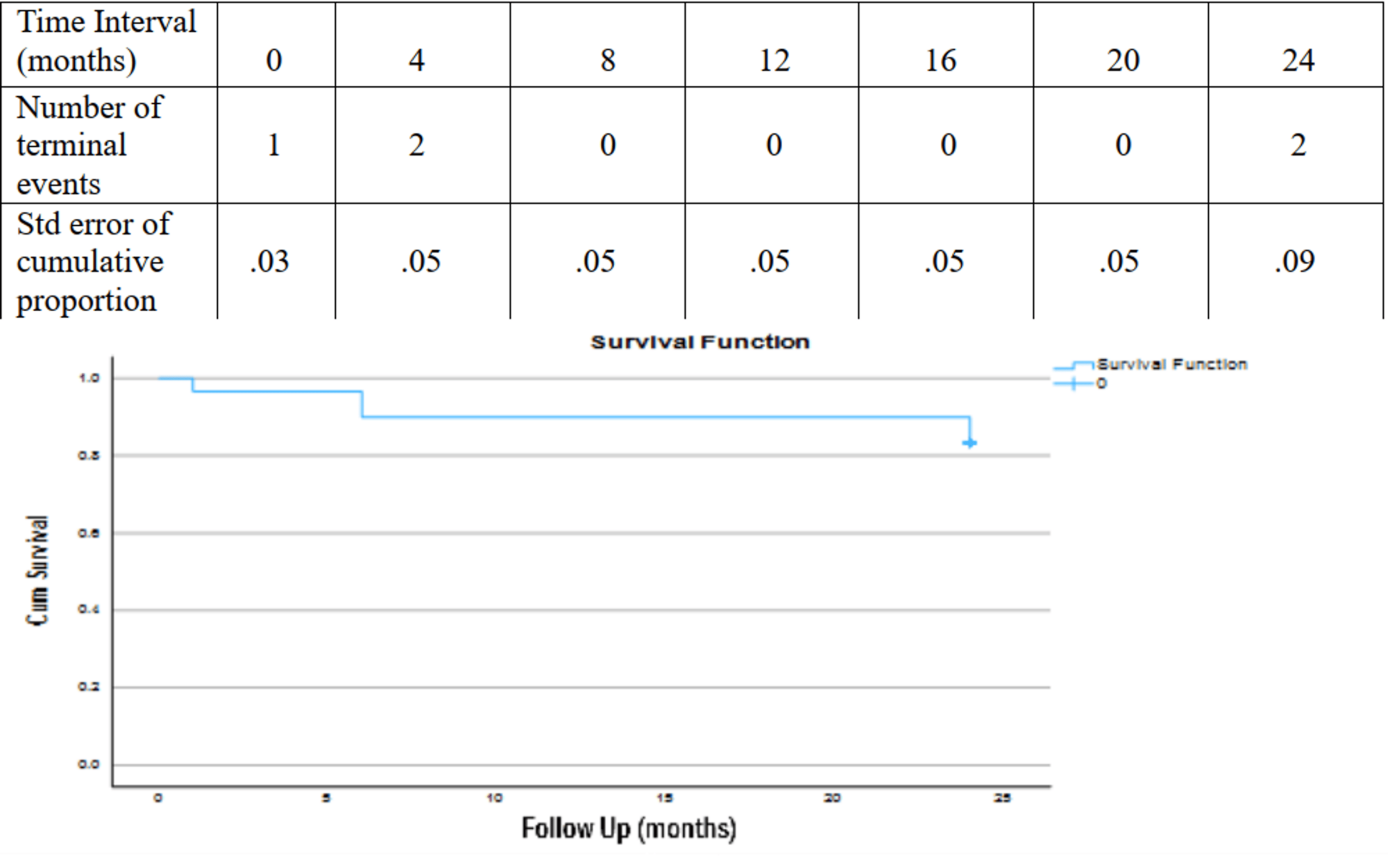
Kaplan-Meier analysis of all-cause mortality in the study group.

The mean duration of follow-up was 22.03 months (range: 1-24 months). No patient was lost. The protocol of both vascular centers included clinical examination at outpatient clinic and computed tomography angiography at one month, six months, one year and then annually postoperatively. All chimney stent grafts and carotid-subclavian bypasses were patent during follow-up. The two intra-operative type Ia ELs vanished at control computed tomography angiography (CTA) at one month. In a subgroup analysis of patients who completed 48 months follow-up (n=8), one patient experienced type Ia endoleak, detected at delayed contrast phase of CTA without increase of the aneurysm sac which was managed conservatively due to severe patient’s comorbidities (heart failure with low ejection fraction and chronic obstructive pulmonary disease). Aortic reintervention rate was 0%. Five patients deceased, all of them were due to ischemic heart disease. All-cause mortality during follow-up was 16.6%. The Kaplan-Meier analysis is presented in Figure 4.

.

## Discussion

Endovascular repair of aortic arch pathologies has gained acceptance as the primary treatment option over the last decade due to low perioperative rates of morbidity and mortality. Parallel graft technique and extra-anatomic bypasses offer the advantages of using financially cheaper off-the-shelf devices, available 24 hours per day in a vascular laboratory, compared to fenestrated and branched endografts. The financial crisis in Greece over the last decade has limited us to use custom-made devices. Many thoracic stent grafts are available in the market, each of them with different technical characteristics to ensure proper apposition to the aortic wall and stability, minimizing endoleaks and distal migration of the endograft. The Gore C-TAG thoracic endoprosthesis (W.L. Gore & Associates, Flagstaff, AZ, United States) is a polytetrafluoroethylene (PTFE) self-expandable stent graft with a new release system without a bare proximal fixation system, permitting partial deployment by 50%, an angulation control system by rotating a dial followed by full deployment of the endograft [12]. The Valiant thoracic stent graft (Medtronic, Inc, Minneapolis, MN, United States) consists of a nitinol skeleton covered by woven polyester graft. It has an eight-peak configuration of the proximal bare spring distributing radial force across more points of contact [13]. The RelayPro thoracic stent graft (Terumo Aortic, Sunrise, Florida) is composed of a nitinol skeleton with polyester synthetic graft. The stents are sutured to the graft fabric for profile reduction. It is available in two proximal end configurations: the bare stent (proximally uncovered stent) and the proximally covered stent. It has a shorter longitudinal curved nitinol wire compared with the previous generation that provides longitudinal support throughout the length of the device and enhances stent-graft conformability to the aortic arch [14]. The Zenith low profile thoracic stent graft (Cook Inc, Bloomington, IN, United States) is a self-expanding nitinol stent with polyester synthetic graft material. The proximal component of the Zenith Alpha device can be straight or tapered and incorporates a bare alignment proximal stent, whereas the precurved cannula on the delivery system is designed to assist in adequate proximal arch conformability [15]. Ankura thoracic stent graft is a novel device which was introduced in Europe the previous decade. Most of the reports in the literature come from China with acceptable perioperative and aortic-related long-term results [10,16,17]. Kratimenos et al. [9] were the first in Europe to evaluate the efficacy and safety of Ankura thoracic stent graft in descending thoracic aortic pathology. The technical success of thoracic endovascular aortic repair (TEVAR) was 97% with 29 of 30 patients achieving successful outcomes. During the follow-up period, there were no aneurysm-related deaths, and the procedure was associated with low rates of endoleaks and overall complications. The Ankura Italian Data Collection Group provided their preliminary experience with in situ fenestration at zone 2 during TEVAR with Ankura endograft. Technical success was 94%, 2.5% mortality (non-aortic related), and no structural issues or need of reintervention during the follow-up [18]. To the best of our knowledge, this is the first report in Europe evaluating perioperative and mid-term efficacy of thoracic Ankura in aortic arch pathologies.

In the present study, technical success was 94%. No migration and no technical issues were noticed during deployment of the graft in this challenging area. The patency of supra-aortic branches treated with chimney technique and extra-anatomic bypass was 100%. Type Ia endoleak is the “Achille’s tendon” of chimney TEVAR in aortic arch, due to gutter between aortic stent, branch stent and aortic wall. In a meta-analysis by Li et al. [19], the pooled technical success rate of chimney technique was 91% (95% confidence interval (CI): 87%-94%), the rate of patency was 93% (95% CI: 89-96%), and the rate of perioperative endoleaks was 21% (95% CI: 17-26%) with only 3.7% of patients required reintervention. This aligns with the results of Ullery et al. [20], that 70% of type Ia endoleaks could resolve in 12 months and only 3.3% of patients required reintervention. In a European multicenter registry, type Ia EL was noticed in 10% of patients intraoperatively, resolving by half during the first 30 days after operation [21]. In our study, two patients had type Ia EL at final angiography, detected at delayed phase and managed conservatively which eventually were subsided at control CTA at one month. This discrepancy with our results could be justified on different types of thoracic endografts used, stent graft oversizing, overlapping of chimney stents and types of covered stents at supra-aortic branches (balloon-expandable/self-expandable). We believe that it is most likely because of the PTFE synthetic graft which provides zero porosity and better flexibility in the aortic arch compared to polyester grafts, better adherence of Ankura endograft to the aortic wall due to the presence of connecting bar along the outer curve of the arch as well as the radial force of the proximal bare stent minimizing gutters. Lachat et al. [22] suggested an overlap at least 2 to 3 cm for chimney stent grafts to minimize gutters and type Ia EL.

Neurological complications such as transient ischemic attacks and stroke are major causes of morbidity and mortality in aortic arch repair. In a meta-analysis by Moulakakis et al. [23], stroke rate in hybrid repair of aortic arch was 7.6%. In the present study, stroke rate was 3.3%. One patient experienced right hemiparesis after awakening probably due to thromboembolism in the presence of a severe atheromatous disease of aortic arch. Nerve injury during carotid-subclavian bypass in the setting of TEVAR is a common adverse event that can cause significant postoperative respiratory, speech and mobility complications. Meticulous dissection of supra-clavicular area, with recognition and protection of vagus nerve, phrenic nerve and brachial plexus, is mandatory to avoid adverse events. Rylski et al. [24] reported results in 118 patients after carotid-subclavian bypass. Postoperative complications included left-sided stroke in 3% and axillary, phrenic, and recurrent laryngeal nerve palsy in 3%, 2%, and 3%, respectively. On the contrary, Voigt et al. [25] reported their results regarding short-term complications after carotid-subclavian bypass in the setting of TEVAR. In 102 patients, phrenic nerve palsy was observed in 27 patients (26.4%), which was extremely high, recurrent laryngeal nerve palsy in six patients (5.8%) and axillary nerve palsy in two patients (1.9%). In the current study, one patient had axillary nerve injury probably due to traction from Weitlaner retractor with minor motor disturbances of the left upper limb.

Limitations

This study has several limitations. It is a retrospective, observational study of two vascular centers, with 30 patients. The study group is heterogeneous, and we do not provide any comparison between hybrid and endovascular techniques of perfusion of supra-aortic branches. The number of patients who received Ankura thoracic stent graft in western countries for aortic arch pathologies is limited in literature to compare our results with other vascular centers.

## Conclusions

Thoracic endovascular repair with Ankura stent graft in conjunction with hybrid and endovascular procedures is a safe and effective endoprosthesis for repair of aortic arch lesions. It showed high technical success, with a low overall endoleak rate and complication rate during follow-up. Larger studies are necessary with a longer follow-up to establish its efficacy.
